# Integrated dual-mode 3 dB power coupler based on tapered directional coupler

**DOI:** 10.1038/srep23516

**Published:** 2016-03-22

**Authors:** Yuchan Luo, Yu Yu, Mengyuan Ye, Chunlei Sun, Xinliang Zhang

**Affiliations:** 1 Wuhan National Laboratory for Optoelectronics, School of Optical and Electronic Information, Huazhong University of Science and Technology, Wuhan, 430074, China

## Abstract

A dual-mode 3 dB power coupler based on silicon-on-insulator platform for mode division multiplexing system is proposed and demonstrated. The device, which consists of a tapered directional coupler and two output bend waveguides, has a 50:50 coupling ratio around the wavelength of 1550 nm for both fundamental and first order transverse magnetic (TM_0_ and TM_1_) modes. Based on asymmetrical tapered structure, a short common coupling length of ~15.2 μm for both modes is realized by optimizing the width of the tapered waveguide. The measured insertion loss for both modes is less than 0.7 dB. The crosstalks are about −14.3 dB for TM_0_ mode and −18.1 dB for TM_1_ mode.

Optical communication technology has made great strides for several decades and the capacity per fiber is gradually approaching the Shannon limit^1^. Fortunately, mode division multiplexing (MDM) provides a new dimension and it has been considered as a potential option to expand on-chip bandwidth by exploiting the high order modes of the multimode waveguides in recent years^2^,^3^. In order to realize a MDM system, many devices, including multiplexer^4^,^5^,^6^,^7^, amplifier^8^ and optical switching^9^,^10^, had been redesigned due to incompatibility with high order modes. The power coupler is an essential component in integrated optical communication systems and is widely used in many applications. However, the power coupler has mainly been achieved in single-mode communication networks, and the efforts are focused on enhancing the bandwidth^11^,^12^,^13^,^14^,^15^,^16^,^17^,^18^ or realizing polarization independent^11^,^14^,^19^, while the investigations on multi-mode dimension have not been reported, to the best of our knowledge.

The simplest design for power coupler comprises of two parallel waveguides with a small gap^17^. According to the coupled-mode theory^20^, it can be extended to handle high-order mode through specifically designing the waveguides. Generally, the power coupler with symmetrical waveguide structure is sensitive to wavelength and coupling length. And it can be improved by employing the asymmetrical structure^21^,^22^. In this paper, we propose and demonstrate an on chip dual-mode 3 dB power coupler based on asymmetrical tapered two-waveguide structure. By optimizing the width of the tapered waveguide, the coupling length is chosen to be the least common 3 dB coupling length for dual modes, and thus equal power splitting can be obtained. Benefitting from the asymmetrical tapered structure, the sensitivity of the coupling length for each mode is relaxed. In consequence, the proposed dual-mode 3 dB power coupler is compact in size and easy to fabricate. The characteristics of the coupler are investigated, both theoretically and experimentally. For demonstration, the coupler is fabricated on 220-nm-high silicon-on-insulator (SOI) platform. The measured insertion loss for both fundamental and first order transverse magnetic (TM_0_ and TM_1_) modes are less than 0.7 dB. The crosstalks are about −14.3 dB for TM_0_ mode and −18.1 dB for TM_1_ mode. The performance of the coupler indicates that it can operate on multiple modes and would find potential applications in on-chip high bandwidth communications.

## Results

### Principle and simulation

**Figure 1:**
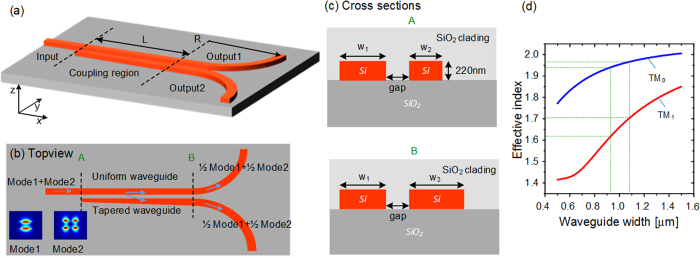
The schematic configuration and the geometric parameters of the proposed dual-mode 3dB power coupler. (**a**) The 3-dimension structure and (**b**) topview of the proposed device. (**c**) The cross sections of the front (**A**) and the end (**B**) of the coupling region in (**b**). (**d**) The simulated effective indices of TM_0_ mode and TM_1_ mode associated with different waveguide widths at 1550 nm.

The schematic configuration of the proposed scheme is illustrated in Fig. 1. The straight coupling region consists of a uniform waveguide and a tapered waveguide to form an asymmetrical directional coupler. Here, the transverse magnetic (TM) mode is utilized. The widths of the two waveguides are designed to support TM_0_ and TM_1_ modes. In order to realize a 50:50 coupling ratio simultaneously, the common 3 dB coupling length of the two modes is selected to be the coupling length. The single-mode couplers based on symmetrical structure are sensitive to wavelength and the coupling length. Considering the high order modes, the sensitivity of wavelength and coupling length will be strengthened, resulting in the common coupling length for two modes will be very long and inapplicable for dual modes design. In our scheme, a tapered waveguide is utilized to alleviated the issue of wavelength sensitivity and relax the precision of coupling length^13^,^22^,^23^. As shown in Fig. 1(b), half energy is coupled from the input waveguide to another waveguide through the coupling region for TM_0_ and TM_1_ modes, resulting in 3 dB power splitting for both modes. The length of the coupling region is defined as L, and other parameters are shown in Fig. 1(c). We use the multimode bend waveguides with radius R to separate the two output ports. The R is chosen to be 25 μm to avoid excess loss while not compromise the size of the device.

**Figure 2:**
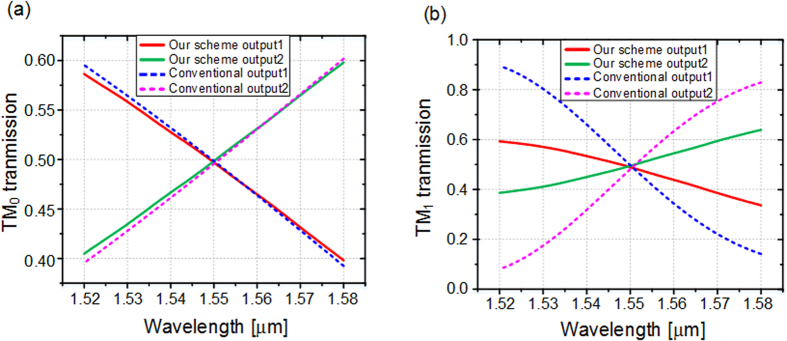
Simulated transmission spectra of our scheme compared with the conventional coupler. The results of (**a**) TM_0_ mode and (**b**) TM_1_ mode input. The conventional coupler for TM_0_ consists of two strip waveguides whose widths are 1.0 μm and heights are 220 nm. The gap between the two waveguides is 200 nm. The parameters for TM_1_ is the same except the coupling length.

**Figure 3:**
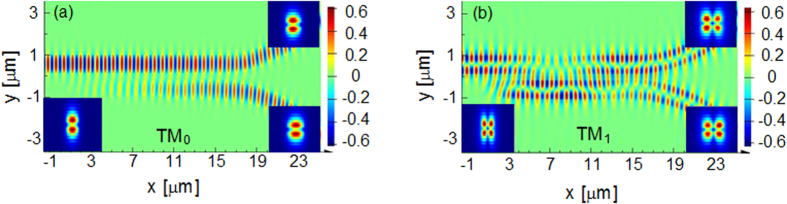
Simulated electric field distribution of Ez component along the propagation direction for dual mode at the wavelength of 1550 nm. (**a**) TM_0_ mode and (**b**) TM_1_ mode. The inserts show the mode distribution of each input and output ports.

**Figure 4:**
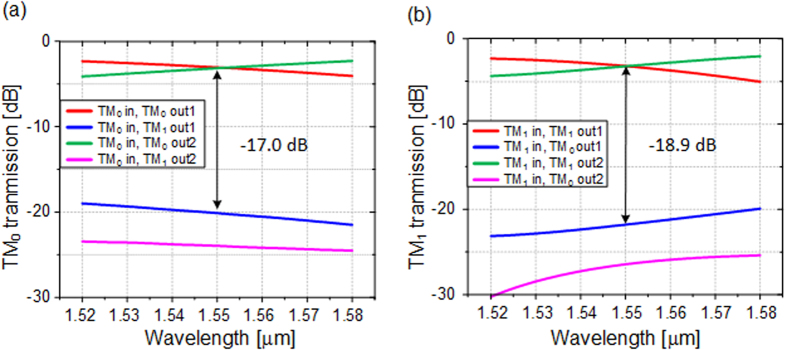
The crosstalk analysis of the device. The power of TM_0_ mode component and TM_1_ mode component at the two output ports when inputting (**a**) TM_0_ mode or (**b**) TM_1_ mode.

**Figure 5:**
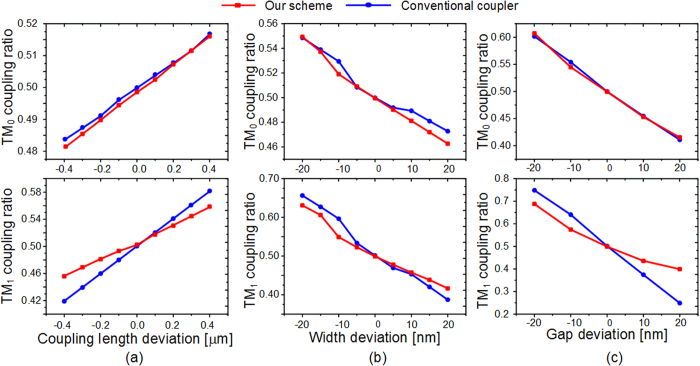
Simulated coupling ratios as a function of geometric parameters deviations for both our scheme and the conventional coulper. The tolerence to the deviation of (**a**) coupling length, (**b**) waveguide width and (**c**) gap. The operation wavelength is 1550 nm.

In the simulation, the width of the uniform waveguide *w*_1_ is chosen to be 1 μm, which can stably support the TM_1_ mode. The gap between the two waveguides is fixed to be 200 nm. The coupling length L, which is the least common 3 dB coupling length of the two modes, is determined based on the optimized result of waveguide width. The width *w*_2_ is supposed to be *w*_1_ − Δ*w* while *w*_3_ is *w*_1_ + Δ*w*. The effective index of TM_0_ mode is almost invariable when the waveguide is tapered around 1 μm, while the one of TM_1_ mode is dramatically changed, as indicated in Fig. 1(d). Therefore, the tapered directional coupler can be regarded as a conventional coupler for TM_0_ mode but a tapered asymmetrical coupler for TM_1_ mode. Hence, the coupling length L can be set as the 3 dB coupling length of TM_0_ roughly, and Δ*w* is optimized to mainly adjust the 3 dB coupling length of TM_1_ mode, targeting to obtain a short common 3 dB coupling length. Considering the two modes simultaneously, the finally optimized width of tapered waveguide varies from *w*_2_ = 0.93 μm to *w*_3_ = 1.08 μm. The optimized coupling length L is 15.2 μm. The final results are presented as the red and green curves in Fig. 2. It is worth noting that the transmission spectra of both modes are almost the same and the coupling ratios are around 50:50 at the wavelength of 1550 nm. The simulated insertion losses of both modes are lower than 0.1 dB which can be neglected. The transmission spectra of conventional symmetrical directional couplers are calculated as a contrast, as shown by blue and pink dash curves in Fig. 2(a,b). One can find that, the coupling ratio dependency on wavelength of our scheme is slightly improved in the case of TM_0_ mode input, and the improvement for TM_1_ mode is obvious. The difference is accessible because the tapered asymmetrical structure mainly works on TM_1_ mode. Figure 3(a,b) present the electric field distributions of TM_0_ and TM_1_ modes along the propagation direction at 1550 nm respectively, while the inserts are the mode distributions of the input and two output ports. The input TM_0_ and TM_1_ modes are evenly divided around the point of L = 15.2 μm. The inserts show that the output mode is the same with the input mode, qualitatively indicating a low crosstalk. Additionally, we utilize the mode expansion method to quantitatively calculate the crosstalk of the whole coupler with the output bend waveguides. The simulated results are shown in Fig. 4. The crosstalks, defined as the ratio of the sum of the interfering mode power to the desired mode power, are −17.0 dB for TM_0_ mode and −18.9 dB for TM_1_ mode. The higher crosstalk of TM_0_ might be caused by the multimode bend waveguides^24^,^25^, which would introduce a crosstalk in transmission and can be reduced by increasing the radius or special design. As mentioned previously, the performance of the directional-coupler-based device is sensitive to geometric parameters. The fabrication tolerances to the deviations of coupling length, waveguide width and gap are investigated, as shown in Fig. 5. The same calculations for conventional scheme are also presented as references. Results indicate improvement on the fabrication tolerance compared with the conventional coupler.

### Experimental results

**Figure 6:**
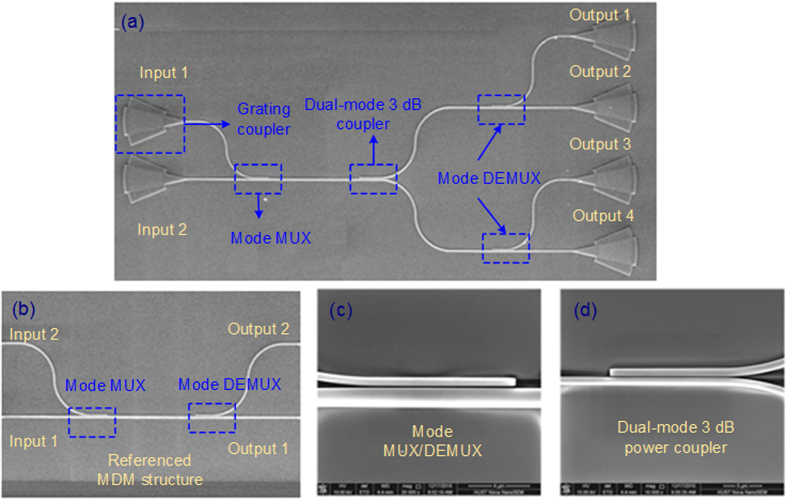
The SEM top view of the fabricated device. (**a**) Test structure consisting of a mode multiplexer, a dual-mode 3 dB power coupler and a mode demultiplexer. (**b**) Referenced MDM structure for calibration, (**c**) the detail of mode MUX/DEMUX and (**d**) dual-mode 3 dB power coupler we proposed.

Figure 6(a) shows the scanning electron micrograph (SEM) of the fabricated structure. Due to the lack of TM_1_ source and other necessary facilities for high order mode, an extra mode multiplexer is designed for TM_1_ signal inputting and another mode demultiplexer is utilized for output TM_1_ measurement. The input TM_0_ mode from Input 1 (marked in Fig. 6(a)) is converted to TM_1_ mode by the multiplexer, and then the converted TM_1_ mode divides evenly into the two output waveguides, which are finally converted to TM_0_ modes and coupled out from the Output 1 and Output 3, respectively. On the other hand, the input TM_0_ mode from the Input 2 propagates straightly to the 3 dB coupler and couples out from the Output 2 and Output 4 after division. In order to calibrate the insertion loss of the device we designed, a referenced MDM structure including a mode multiplexer and a demultiplexer is also fabricated, as shown in Fig. 6(b). The detailed SEM of the mode multiplexer is shown in Fig. 6(c). The mode multiplexer is based on asymmetrical directional coupler. The TM_0_-TM_1_ coupling relies on the phase matching between the waveguides, which means that the effective index of the TM_0_ mode of the narrow waveguide should be equal to that of the TM_1_ mode of the wide waveguide. The optimized width of the single-mode and multimode waveguides are 0.5 μm and 1.23 μm respectively, while the coupling length is 9.5 μm. Figure 6(d) shows the detail of the fabricated dual-mode 3 dB power coupler. The focusing grating couplers are used to couple TM_0_ mode into and out of the chip^26^,^27^.

**Figure 7:**
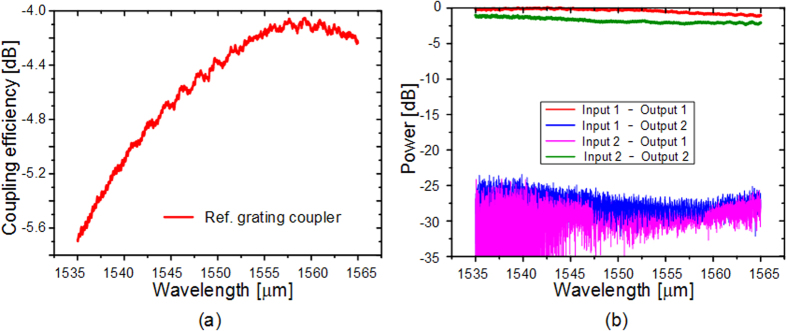
The performance of the referenced grating coupler and MDM structure. (**a**) The measured coupling efficiency of the grating coupler. (**b**) The experimental results of the mode multiplexer. The definitions of the MDM ports are marked in Fig. 6(b).

**Figure 8:**
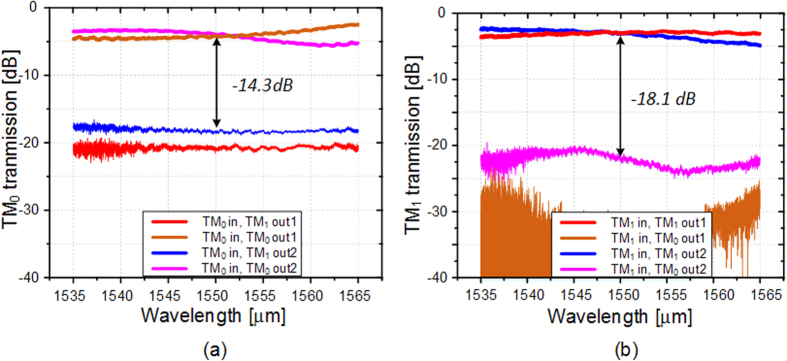
The optical performance of the fabricated device. The measured transmission and crosstalk at the four output ports of the test structure when inputting (**a**) TM_0_ mode and (**b**) TM_1_ mode.

To evaluate the performance of the proposed device, a broadband light source assisted by the polarization controller and an optical spectrum analyzer (OSA) are used to measure the transmission spectra of the device. Figure 7(a) presents the measured coupling efficiency of the grating coupler. The coupling loss is about 4.3 dB for one port at 1550 nm. The experimental results of the mode multiplexer are shown in Fig. 7(b). The excess losses are about 0.2 dB and 2.1 dB for TM_0_ and TM_1_ mode channels, respectively. Low crosstalks of −25 dB for the two mode channels are observed. The average transmission spectra of TM_0_ and TM_1_ are shown in Fig. 8(a,b), respectively. The insertion loss introduced by the coupling gratings and the MDM structure have been deducted. The experimental results almost conform to the simulated ones shown in Fig. 2(a,b). The insertion loss of the TM_0_ mode is ~ 0.7 dB, while the one of TM_1_ mode is ~0.2 dB. The difference is caused by the inconformity between the MDM structure used in the test structure and the referenced one, owing to the imperfect fabrication. The measured crosstalks for TM_0_ and TM_1_ transmissions are −14.3 dB and −18.1 dB, respectively.

## Discussion

In conclusion, we propose and design an on-chip dual-mode 3dB power coupler using an asymmetrical directional coupler built on the SOI platform. The device can be easily fabricated in a single step of exposure and etching. Simulated and experimental results show an even division for both TM_0_ and TM_1_ modes at the wavelength of 1550 nm, with insertion losses of about 0.7 dB and 0.2 dB. The measured crosstalks for TM_0_ and TM_1_ modes are about −14.3 dB and −18.1 dB, respectively. The fabrication tolerances to the deviations of coupling length, waveguide width and gap are also discussed, and the results show improvement compared with the conventional coupler. The coupler can accommodate the mode division multiplexed system and would be applied in future silicon based large capacity communication systems.

## Methods

### Simulation method

We use a three-dimension finite difference time domain (FDTD) method to simulate the proposed dual-mode 3 dB coupler. In order to save the simulation time, we firstly optimize the performance of the coupler without the two bend waveguides to obtain the rough parameters. It is to be noted that, there is still a small coupling in the bend region, which is not considered in the simulation. So in the next step, we optimize the performance of the whole coupler by making a small change around the rough parameters.

### Device fabrication and experimental method

The device is fabricated utilizing 248 nm deep ultraviolet photolithography and inductively coupled plasma (ICP) etching using SOI wafer with top silicon layer of 220 nm and silicon dioxide (SiO_2_) substrate of 2 μm. The etched structures have a SiO_2_ cladding layer by utilizing plasma-enhanced chemical vapor deposition. Due to the lack of TM_1_ source and other necessary facilities for high order mode, an extra mode multiplexer is designed for TM_1_ signal inputting and another mode demultiplexer is utilized for output TM_1_ measurement. In order to calibrate the insertion loss of the device we designed, a referenced MDM structure including a mode multiplexer and a demultiplexer is also fabricated. Since the results are easily disturbed by a lot of factors, we test the device many times and average the results, aiming to reduce the measuring error and alleviate the influence introduced by environment.

### Crosstalk calculation

We employ the mode expansion method to analyze the crosstalk of the dual-mode power coupler in the simulation. The mode expansion method is to calculate the power proportion of each mode inside one waveguide based on overlap integral, as explained by following formula:


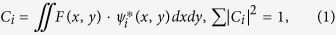


where the 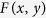 is the input mode field and the 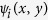 is a set of modes that the waveguide supports.

## Additional Information

**How to cite this article**: Luo, Y. *et al*. Integrated dual-mode 3 dB power coupler based on tapered directional coupler. *Sci. Rep.* **6**, 23516; doi: 10.1038/srep23516 (2016).
